# Post-radiation xerostomia therapy with allogeneic mesenchymal stromal stem cells in patients with head and neck cancer: study protocol for phase I clinical trial

**DOI:** 10.2478/raon-2023-0052

**Published:** 2023-11-30

**Authors:** Primoz Strojan, Gaber Plavc, Marko Kokalj, Goran Mitrovic, Olga Blatnik, Luka Lezaic, Aljaz Socan, Aljosa Bavec, Natasa Tesic, Katrina Hartman, Urban Svajger

**Affiliations:** Institute of Oncology Ljubljana, Ljubljana, Slovenia; University of Ljubljana, Faculty of Medicine, Ljubljana, Slovenia; University Medical Centre Ljubljana, Department of Nuclear Medicine, Ljubljana, Slovenia; Blood Transfusion Center of Slovenia, Ljubljana, Slovenia; University of Ljubljana, Faculty of Pharmacy, Ljubljana, Slovenia

**Keywords:** oropharyngeal cancer, xerostomia, mesenchymal stromal stem cells

## Abstract

**Background:**

Xerostomia is a common side effect of radiotherapy in patients with head and neck tumors that negatively affects quality of life. There is no known effective standard treatment for xerostomia. Here, we present the study protocol used to evaluate the safety and preliminary efficacy of allogeneic mesenchymal stromal stem cells (MSCs) derived from umbilical cord tissue.

**Patients and methods:**

Ten oropharyngeal cancer patients with post-radiation xerostomia and no evidence of disease recurrence 2 or more years after (chemo)irradiation (intervention group) and 10 healthy volunteers (control group) will be enrolled in this nonrandomized, open-label, phase I exploratory study. MSCs from umbilical cord tissue will be inserted under ultrasound guidance into both parotid glands and both submandibular glands of the patients. Toxicity of the procedure will be assessed according to CTCAE v5.0 criteria at days 0, 1, 5, 28, and 120. Efficacy will be assessed by measuring salivary flow and analyzing its composition, scintigraphic evaluation of MSC grafting, retention, and migration, and questionnaires measuring subjective xerostomia and quality of life. In addition, the radiological, functional, and morphological characteristics of the salivary tissue will be assessed before, at 4 weeks, and at 4 months after the procedure. In the control group subjects, only salivary flow rate and salivary composition will be determined.

**Discussion:**

The use of allogeneic MSCs from umbilical cord tissue represents an innovative approach for the treatment of xerostomia after radiation. Due to the noninvasive collection procedure, flexibility of cryobanking, and biological advantages, xerostomia therapy using allogeneic MSCs from umbilical cord tissue may have an advantage over other similar therapies.

## Introduction

### Xerostomia in patients with head and neck cancer

Radiotherapy (RT) is one of the main therapeutic modalities for head and neck cancer (HNC): approximately 80% of patients diagnosed with this cancer undergo RT.^1^ Unfortunately, RT is associated with significant toxicity in the head and neck region. While the acute side effects of RT usually occur within 90 days of treatment initiation, the toxic late effects can occur months or even years after treatment completion. Acute side effects occur most frequently in tissues with high proliferation activity, and pathological mechanisms include radiation-induced death of stem cells with the subsequent inflammatory response. Healing and regeneration of the tissue depends on the survival of the remaining stem cells at the site of injury or on their migration from the surrounding tissue, which is less irradiated or not irradiated at all, to the damaged area.^2^ In the head and neck region, the most common RT-related acute side effects include radiodermatitis and radiomucositis, affecting approximately 80% of patients. They also frequently suffer from dysgeusia, dysphagia, and xerostomia, i.e., a feeling of dry mouth due to saliva deficiency or changes in saliva composition.^3^

The first symptoms of salivary deficiency may manifest after the first irradiation fractions and often progress to a chronic condition, especially when the average dose received by the major salivary glands, such as the parotid glands, reaches or exceeds 26 Gy or, in the case of the submandibular glands, 35 Gy.^4^ Unfortunately, despite various improvements and advances, prevention of xerostomia remains a challenge. Modern radiation techniques, such as intensity-modulated radiotherapy, the use of proton or heavy ion beams, allow efficient, although not complete, sparing of the healthy tissue around the tumor.^5,6^ Careful oral hygiene, regular rinsing of the mouth, adequate hydration, and the use of intraoral stents that separate healthy tissue from the tumor are among the generally recommended preventive measures.^7^ The use of amifostine, a free radical scavenger, with its questionable long-term efficacy and side effects occurring in nearly half of patients, remains the subject of professional debate.^8^ Surgical relocation of the submandibular gland contralateral to the tumor to the nonirradiated submental area is an invasive procedure and only suitable for a limited number of HNC patients.^9^

Various synthetic saliva substitutes based on methylcellulose are used to relieve the sensation of dry mouth, but with very variable effects in patients. The same is true for acupuncture, electrical nerve stimulation, and hyperbaric oxygenation.^10^ If salivary gland function is partially preserved, cholinergic analogs (e.g., pilocarpine) can be used to stimulate saliva production, but they are rarely used in practice because of numerous side effects.^10^

Because they do not target regeneration of damaged glandular tissue, existing preventive and therapeutic approaches for newly diagnosed HNC patients or for patients with previously developed radiation-induced salivary gland hypofunction are limited and of questionable efficacy. As mentioned above, the pathophysiology of these complications is multifactorial and includes acute and late processes that may persist for several years after RT. They are characterized by chronic inflammation, development of fibrosis, loss of local stem and progenitor cells, and deterioration of survival conditions for the remaining acinar cells.^4^ Consequently, salivary gland hypofunction and resulting xerostomia remain the most common long-term side effects of RT in HNC patients, which usually has a strong negative impact on their quality of life.^11^

### Mesenchymal stromal stem cells

Mesenchymal stromal stem cells (MSCs) originate from the mesodermal layer from which various mesenchymal tissues develop during embryonic development. In adults, MSCs represent a heterogeneous cell population that includes multipotent MSCs that can differentiate into cartilage, bone, and fat cells. Because of their regenerative capabilities, they are often referred to as “adult stem cells” and are frequently studied in the field of regenerative medicine.^12^ The most common biological sources for MSC production are bone marrow, adipose tissue, umbilical cord tissue, and others. The production and cultivation of MSCs is a relatively straightforward and safe process without significant ethical concerns. MSCs are characterized by low or no expression of major histocompatibility complex molecules, making them suitable for an allogeneic setting. Another phenotypic feature is the simultaneous expression of several markers, including CD73, CD90, and CD105, and the lack of expression of hematopoietic markers such as CD45.^13^

Although a specific subset of MSCs can differentiate into different cell types, there is increasing evidence that the main therapeutic effects of MSCs are mediated by paracrine mechanisms involving the secretion of various active biomolecules. Factors secreted by MSCs may have angiogenic, anti-apoptotic, regenerative, and immunomodulatory effects.^14^ Among all types of stem cells, MSCs possess a unique immunomodulatory effect. Their activity is based on the expression of immunomodulatory enzymes such as indoleamine 2,3-dioxygenase (IDO) and inducible nitric oxide synthase (iNOS), immunosuppressive biomolecules such as prostaglandin E2 (PGE2) and hepatocyte growth factor (HGF), the production of immunosuppressive cytokines such as interleukin (IL)-10 and transforming growth factor (TGF)-β, or the expression of inhibitory surface molecules such as HLA-G and programmed death ligands PD -L1 and PD -L2. With such an extensive repertoire of immunomodulatory mechanisms, MSCs can regulate the function of different types of immune cells belonging to both innate and adaptive immunity. These include monocytes/macrophages as well as important antigen-presenting cells such as dendritic cells (DCs). MSCs can also suppress the activity of T and B lymphocytes, natural killer (NK) cells, neutrophils, and mast cells. On the other hand, MSCs have been shown to promote the generation of regulatory immune cells such as regulatory T cells (Tregs), tolerogenic DCs, and myeloid-derived suppressor cells.^15^ MSCs markedly inhibit T cell proliferation both in an allogeneic setting and upon exposure to various mitogens.^16,17^ In co-cultures with helper Th1-type T cells, MSCs can greatly reduce the secretion of pro-inflammatory cytokines (e.g., IFN-γ and TNF-α) and increase the proportion of T cells producing anti-inflammatory cytokines (e.g., IL −10), such as Tr1 cells.^18^ They may also inhibit the activation of CD8+ cytotoxic T cells, resulting in a decreased response to alloantigens and decreased antigen-specific lysis of allogeneic cells.^19,20^ In summary, MSCs can suppress immune activation and promote a tissue regeneration through mechanisms involving the production/expression of various cell-bound and soluble factors.

An important issue in the clinical translation of MSC-based advanced therapy medicinal products (ATMPs) is the known heterogeneity of MSC preparations used in clinical trials. Variations in final product formulations may result from donor variability, tissue source variability, frequent and extensive cell passage variability during manufacturing, and variable use of fresh or directly thawed cryopreserved MSCs, the suboptimal functionality of which has been reported.^21,22^ In the present study, we will address these issues by introducing a novel protocol for the preparation of MSCs that allows for equivalent final product formulations for each recruited patient by using only freshly cultured cells with very low passages (p ≤ 2) and high viability, as described below.

Advanced therapy medicinal products based on MSCs also represent an interesting and novel therapeutic option for patients with radiation-induced salivary gland damage and existing xerostomia because they act through different mechanisms that have both immunomodulatory and regenerative effects.^23^ Since xerostomia significantly limits patients’ quality of life, exploring the potential efficacy of MSC-based therapy is of high priority and with direct clinical relevance.^11^

## Methods and design

**FIGURE 1.**
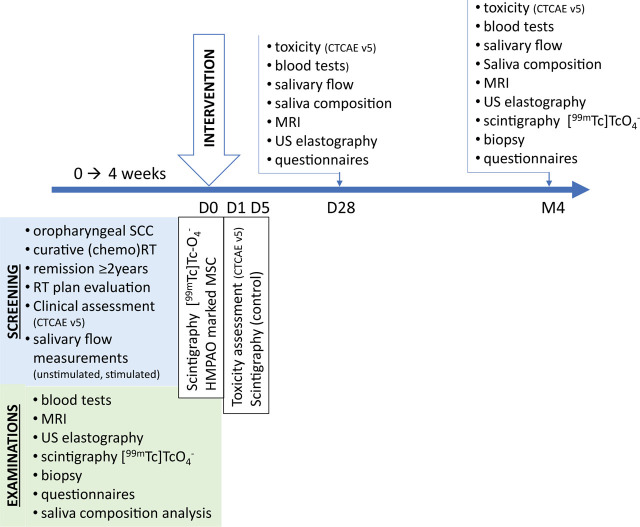
Design of the clinical trial. CTCAE v5 = Common Terminology Criteria for Adverse Events version 5.0; MRI = magnetic resonance imaging; MSC = allogeneic mesenchymal stromal stem cells; RT = radiotherapy; SCC = squamous cell carcinoma; US = ultrasound; [99mTc]*HMPAO* = Technetium 99m-hexamethylpropyleneamine oxime; [99mTc]TcO_4_ = Technetium 99m-pertechnetate

The study is being conducted at the Institute of Oncology Ljubljana in collaboration with the Slovenian Institute for Transfusion Medicine, the Clinic of Nuclear Medicine at the University Medical Centre Ljubljana, and the Institute of Biochemistry and Molecular Genetics at the Faculty of Medicine, University of Ljubljana. It is a non-randomized, single-center, open-label, phase I exploratory study. The study will include 10 patients (intervention group) and 10 healthy volunteers (control group) (Figure 1).

The aim of the study is to evaluate the safety and preliminary efficacy of treatment of xerostomia after irradiation with allogeneic MSC from umbilical cord tissue. Thus, we hypothesize that the treatment of post-radiation xerostomia with allogeneic MSC from umbilical cord tissue is safe and effective.

The study protocol was approved by the National Medical Ethics Committee (No. 0120-193/2023/3) and registered in the ClinicalTrial.gov database (NCT06012604) under the title: “*Treatment of Post-radiation Xerostomia with Allogeneic Mesenchymal Stromal Stem Cells: A Pilot Study”,* registered on 22 August 2023; https://classic.clinicaltrials.gov/ct2/show/NCT06012604

### Objectives

The primary objective of the study is to evaluate the safety of administering allogeneic MSCs to the submandibular and parotid glands of patients with radiation-induced salivary gland dysfunction and xerostomia, with a 4-month follow-up after the procedure.

Secondary objectives of the study include evaluation of the efficacy of the procedure and radiologic, functional, and morphologic changes in glandular tissue after application of allogeneic MSC (Table 1).

**TABLE 1.** Primary and secondary objectives of the study

**Objective**	**Definition of objective**	**Time of evaluation**
**Primary**		
Evaluation of the safety of administration of allogeneic MSCs	Adverse event assessment (CTCAE v.5 criteria): pain at application site, mouth sensation, infection	From the start of therapy to the last follow-up (days 1, 5, 28, and 120)
**Secondary**		
Efficacy of the procedure	Measurement of unstimulated/stimulated salivary flow and saliva composition	During recruitment, day 28 and 120 post-procedure
	Assessment of subjective degree of xerostomia (questionnaires)	During recruitment, days 28 and 120 after the procedure
		Immediately after the procedure (day 0)
	Scintigraphic evaluation of grafting, retention, and migration of allogeneic MSCs	During recruitment, on days 28 and 120 after the procedure
	Assessment of patients’ quality of life	
Radiological changes of the salivary tissue	Magnetic resonance imaging (MRI): volume, signal, and diffusivity changes	During recruitment, on days 28 and 120 after procedure
	Ultrasonography (US): consistency (firmness)	
Functional changes of the salivary tissue	Scintigraphy with ([99mTc]TcO_4_^−^ (pertechnetate): uptake of radioisotope in parenchyma, ejection fraction	During recruitment, on days 28 and 120 after procedure
Morphological changes of the salivary tissue	Core needle biopsy specimens: composition, inflammatory infiltrate, metaplasia, reactive changes	During recruitment, on day 120 after the procedure

CTCAE v.5 = Common Terminology Criteria for Adverse Events version 5.0; MSCs = allogeneic mesenchymal stromal stem cells

### Eligibility criteria

Patients who were successfully treated with (chemo)radiotherapy for squamous cell carcinoma of the oropharynx >2 years ago and have grade 2 or 3 xerostomia after radiotherapy according to the Common Terminology Criteria for Adverse Events (CTCAE) v.5.0 scale will participate in the study.^24^ Inclusion and exclusion criteria will be used to determine whether they can be included in the study (Table 2).

**TABLE 2.** Inclusion and exclusion criteria

**Inclusion criteria**	**Exclusion criteria**
Squamous cell carcinoma of the oropharynx, UICC TNM (8^th^ ed.) stage cT1–2N+ or cT3–4cN0–3 M0, treated with curative intent RT (TD 66–70 Gy, bilateral neck irradiation) with or without concurrent chemotherapy	Newly diagnosed malignant tumor anywhere in the body within the last two years
Two years or more after treatment with no evidence of locoregional recurrence or systemic metastasis	Active smoker
Nonsmoker or former smoker (quit smoking ≥ 2 years ago)	Use of xerogenic medications (e.g., tricyclic antidepressants, antipsychotics, decongestants, bronchodilators, antihypertensive agents such as beta blockers and diuretics, antihistamines, hypnotic sedatives, opioids, and muscle relaxants)
Mean radiation dose > 26 Gy to each of the parotid glands and > 35 Gy to each of the submandibular glands	Other salivary gland disorders (e.g., Sjoegren's syndrome, scleroderma, sialolithiasis, etc.)
Grade 2 or 3 xerostomia as assessed by the CTCAE v5.0 scale	Patients receiving anticoagulant therapy that cannot be discontinued during the procedure
Clinically decreased salivation and hyposalivation (unstimulated total salivary flow of 0.05–0.20 ml/min)	Pregnancy or planned pregnancy within the next two years
Age between 18–75 years	Breastfeeding
Both sexes	Active, uncontrolled infection
Signed “Informed Consent Form” to participate in the study	Other medical (including psychiatric) conditions that, in the opinion of the investigators, preclude safe administration of the planned therapy and completion of follow-up visits
	Known substance abuse or alcoholism

CTCAE v.5 = Common Terminology Criteria for Adverse Events version 5.0; RT = radiotherapy: TD = tumor doses; UICC = *Union for International Cancer Control*

Criteria for subsequent exclusion of patients from the study are: pregnancy, infection at the graft site, allergy to local anesthetics or citric acid, and withdrawal of consent to participate in the study.

If a patient is excluded from the study prior to evaluation of the effect of the intervention under study, she or he will be replaced by another patient, leaving the final number of included patients at 10.

### Inclusion of patients

Candidates for inclusion in the study will be selected from patients who are regularly monitored in the follow-up clinics for patients with head and neck cancer at the Institute of Oncology Ljubljana. Patients who meet the basic inclusion criteria will be informed about the study and invited to participate. The study will not be randomized.

### Pre- and post-treatment assessment

After being informed about participation in the study and signing the Informed Consent form, patients will undergo the following tests and complete the following questionnaires during the 4-week period before MSC application, i.e., the procedure (Table 3). The same measurements will be performed and questionnaires completed at both the 4-week and 4-month follow-up, with the exception of core needle biopsy and salivary gland scintigraphy (these will be repeated only at the 4-month follow-up). All procedures in the study are listed in Table 3.

**TABLE 3.** Procedures of the study

**Procedures**	**Inclusion**	**Intervention**	**Follow-up examinations**			

	**W4→0**	**D0**	**D1**	**D5**	**D28**	**D120**
Patient screening with clinical and objective evaluation of xerostomia^1^	X					
Complete blood count, biochemistry	X				X	X
Coagulation profile	X					
Measurement of unstimulated and stimulated salivary flow rate	X				X	X
Determination of salivary composition	X				X	X
Magnetic resonance imaging	X				X	X
US elastography	X				X	X
Scintigraphy with [99mTc]Tc-HMPAO labeled MSCs		X				
Scintigraphy with (*[99mTc]TcO_4_^−^*)	X					X
Core needle biopsy of the gland^2^	X					X
Visual Analog Scale questionnaire	X				X	X
Xerostomia Questionnaire	X				X	X
EORTC QLQ-H&N35	X				X	X
Toxicity assessment, CTCAE v.5		X	X	X	X	X

CTCAE v.5 = Common Terminology Criteria for Adverse Events version 5.0; D = day; EORTC QLQ-H&N35 = European Organisation for Research and Treatment of Cancer Quality of Life Questionnaire Head and Neck Module; MSCs = allogeneic mesenchymal stromal stem cells; US = ultrasound; W = week; [99mTc]*HMPAO = T*echnetium 99m-hexamethylpropyleneamine oxime; *[99mTc]TcO_4_^−^* = *T*echnetium 99m-pertechnetate

1 Inclusion criteria, see Table 2.

2 From the parotid gland: the first 5 patients included in the study; from the submandibular gland: the last 5 patients included. The gland on the side that received a higher dose of radiation will be punctured.

The study also involves 10 volunteers (control group) who do not participate in the procedure. They will sign the Informed Consent to Participate in the Study and provide saliva samples for the measurement of unstimulated and stimulated salivary flow and for the determination of salivary composition (for comparison with the intervention group).

#### Measurement of unstimulated total salivary flow rate

Patients will be instructed to drink at least 2 liters of fluid the day before saliva sample delivery and to abstain from food, drink, and oral hygiene for at least 1 hour before. After a 5-minute break, they will rinse the mouth with a sip (15–20 ml) of cold water from the refrigerator. Unstimulated saliva will be collected for 10 minutes in a pre-weighed disposable plastic container. Saliva samples will be frozen in liquid nitrogen 15 minutes after collection and stored at −80°C for further analysis. Saliva collection will take place between 10:00 and 12:00 in the same room and in the presence of the same physician, before the procedure, 4 weeks and 4 months after the procedure. The flow rate will be calculated assuming that 1 g of saliva is equivalent to 1 ml.^25^

#### Measurement of stimulated total salivary flow rate

The procedures before and after saliva collection will be the same as those for measuring unstimulated saliva. After a 5-min break, patients will chew tasteless kerosene wax for 1 min and rinse the mouth with a sip (15–20 ml) of cold water from the refrigerator. Stimulated saliva collection (while chewing the wax) will be performed for 5 minutes in a disposable plastic container, before the procedure, at 4 weeks, and at 4 months after the procedure.

#### Analysis of the composition of unstimulated and stimulated saliva samples

Several characteristics or components will be determined in the saliva samples:

- pH measurement (SevenCompact™ pH meter S210 with the electrode InLab Micro Pro-ISM, Mettler Toledo, Columbus, Ohio);
- total protein concentration, spectrophotometry at 280 nm (NanoDrop One, ThermoFisher Scientific Inc., Waltham, MA, USA);
- α-amylase activity, spectrophotometry at 405 nm (GENESYS™ 150 Vis/UV, ThermoFisher Scientific Inc., Waltham, MA, USA);
- concentration of mucins (MUC1), ELISA (ThermoFisher Scientific Inc., Waltham, MA, USA);
- total esterase activity, spectrophotometry at 270 nm (GENESYS™ 150 Vis/UV, ThermoFisher Scientific Inc., Waltham, MA, USA)^26^;
- paraoxonase activity, spectrophotometry at 405 nm (GENESYS™ 150 Vis/UV, ThermoFisher Scientific Inc, Waltham, MA, USA).^27,28^

#### Contrast-enhanced magnetic resonance imaging (MRI) of the neck

MRI scans will be performed before, 4 weeks, and 4 months after MSC application using a 1.5T General Electric Optima 450W scanner. The protocol will include T1 FSE sequences in the axial plane, T2 PROPELLER sequences, diffusion weighted sequences (b=0, b=400, b=800) with calculation of ADC maps and 3D T1 SPGR sequences. Volumetric analysis of salivary glands using AW server software, signal changes and diffusivity analysis will be performed.

#### Ultrasonographic examination of the salivary glands

Ultrasonography elastography (UE) will be performed before, 4 weeks, and 4 months after MSC application to assess the consistency (firmness) of the parotid and submandibular glands. Measurements will be taken using the Hitachi Arrieta 850 device immediately prior to ultrasound-guided biopsy of one of the glands.

#### Scintigraphy with [99mTc]Tc-HMPAO-labeled MSCs.

One hour and twenty-four hours (the next day) after MSC application, planar head and whole-body images and SPECT/CT images of the head will be performed to assess the distribution, retention, and migration of MSCs from the application site.

#### Scintigraphy with *[99mTc]TcO_4_^−^*

Before and 4 months after MSC transplantation, scintigraphy of the salivary glands will be performed with the radiopharmaceutical pertechnetate (*[99mTc]TcO_4_^−^*). After intravenous administration, the radiopharmaceutical accumulates in the functioning parenchyma of the salivary glands and is subsequently excreted into the oral cavity after stimulation with citric acid. After administration of the radiopharmaceutical, a 10-minute planar head recording will be made, followed by oral administration and stimulation of the salivary glands with citric acid and another 10-minute planar recording. In this study, the uptake of the radiopharmaceutical in the functioning parenchyma and the excretory fraction of each gland will be evaluated qualitatively and semiquantitatively. Both parameters, determined before and after MSC transplantation will be compared.

#### Percutaneous core needle biopsy of the salivary gland.

A sample will be taken from the parotid gland in the first 5 patients enrolled in the study, while a sample will be taken from the submandibular gland in the last 5 patients. The gland on the side of the head or neck that received a higher dose of radiation during RT will be punctured. The biopsy will be performed in each patient before the procedure and 4 months after, both times from the same gland.

The biopsy will be performed under local anesthesia and ultrasound guidance. Before the procedure, blood coagulation parameters will be determined (platelet count, prothrombin time, international normalized ratio). Patients taking anticoagulant medications will be instructed to discontinue them one week prior to the procedure.

Tissue samples stained with hematoxylin and eosin will be examined by a pathologist. For both pre- and post-procedure specimens, the pathologist will semi-quantitatively evaluate and compare the percentage of serous, mucinous, and mixed acini, ducts, adipose tissue, and fibrosis in the specimen. The pathologist will also evaluate the presence, amount, and composition of the inflammatory infiltrate, possible metaplasia (e.g., squamous or oncocytosis), and reactive changes such as hyperplasia, atrophy, and signs of regeneration. Any other pathologic findings will also be described. If necessary and if sufficient tissue will be available, additional special and immunohistochemical staining will be performed.

#### Questionnaires for subjective assessment of xerostomia

Patients will complete the Visual Analog Scale (VAS) questionnaire and the Xerostomia questionnaire, before the procedure, at the follow-up visits at 4 weeks and 4 months after the procedure.^29,30^ The VAS questionnaire consists of 8 questions that assess two basic aspects of salivary secretion: mucosal dryness and functional abilities (swallowing, speaking) resulting from mucosal dryness. Patients are asked to mark their response on a 100-mm horizontal line for each question.^29^ The Xerostomia questionnaire also consists of eight questions: the first four questions refer to dryness of the mucous membranes during eating or chewing, whereas the last four questions refer to situations in which the person does not eat or chew. Patients rate each symptom on an 11-point Likert scale from 0 to 10, with a higher number indicating more pronounced dryness or greater discomfort due to lack of saliva.^30^

#### Quality of life assessment questionnaire

Patients will complete the European Organization for Research and Treatment of Cancer (EORTC) Quality of Life Questionnaire Head and Neck Module (QLQ-H&N35) before procedure and at follow-up visits at 4 weeks and 4 months after procedure. This questionnaire consists of 35 questions divided into seven domains and 11 individual questions. The questionnaire assesses symptoms and side effects of head and neck cancer treatment, social integration, and sexual functioning. Questions are rated using a four-point Likert scale, except for the last five questions, which offer two response options (yes/no).^31^

#### Blood tests

Venous blood samples will be collected before the procedure and at the follow-up visits 4 weeks and 4 months after the procedure, following the blood collection protocol used at the Institute of Oncology Ljubljana. The values of standard parameters of complete blood count, differential blood count, liver function tests, as well as electrolyte levels, renal markers, total proteins, albumin and C-reactive protein will be analyzed. Prothrombin time and international normalized ratio will also be determined during the first blood draw.

## Intervention

MSC-based investigational medicinal products (IMPs) will be prepared at the Slovenian Institute for Transfusion Medicine according to validated standard operating procedures approved by the National Medical Ethics Committee (No. 0120-60/2018/7, 24.4.2018). The umbilical cord tissue will be used as the primary biological source. The tissue is first seeded in an *ex vivo* culture. After the first 10–14 days (passage 0), adherent cells begin to proliferate extensively and reach adequate confluence within the next 7 days. They are then harvested in passage 0 and reseeded for further cell expansion (passage 1). At the end of passage 1, cells from 4 different donors are harvested and pooled into a single cell suspension in equal ratios. They are then redistributed into identical aliquots and cryopreserved as “off-the-shelf units” (EQ-MSC) available for “per patient” orders. For each patient, an identical aliquot of EQ-MSCs is thawed and seeded in cell culture for 3–5 days prior to the procedure (passage 2) to achieve optimal numbers and cell fitness for the final product. The final drug formulation will be prepared as a suspension of 50×10^6^ MSCs/ml in a physiological solution with an addition of 0.5% human albumin. It will be filled in syringes of 0.5 ml or 1.0 ml with a 23G 0.6×25 mm hypodermic needle.

In the intervention group, each patient will receive an ultrasound-guided injection of 50×10^6^ MSCs into each parotid gland and an injection of 25×10^6^ MSCs into each submandibular gland, without anesthesia. To ensure even distribution of the MSC suspension, each parotid gland will receive injections in two areas: the tail and the body of the gland.

Approximately 5% of the volume of the prepared MSC injectate will be removed under aseptic conditions. The cells will be labeled with hexamethylpropyleneamine oxime ([99mTc]Tc-HMPAO) and resuspended with the remaining injection material for administration to the patient.

### Course of the study

After the procedure (day 0), patients (intervention group) will be examined at day 1, day 5, after 4 weeks, and after 4 months. An assessment of toxicity will be performed at each visit. At 4 weeks and 4 months after the intervention, the same measurements and questionnaires will be performed as when patients were enrolled in the study, except for core needle biopsy and scintigraphy, which will be repeated only at 4 months (Figure 1).

No follow-up is scheduled for the volunteers (control group) after enrollment in the study and the initial examinations (day 0).

The study will continue until completion of follow-up for the last enrolled patient or for a maximum of two years. Tentatively, the study is expected to run from October 2023 to September 2025.

### Termination guidelines

The study will be terminated early if serious adverse events are noted during the intervention or follow-up. An allergic reaction, injection site infection, or other local or systemic event assessed as possibly/probably/surely related to the intervention and graded according to CTCAE v5.0 grade 3 or higher (serious adverse reaction, SAR) will be considered a discontinuation criterion. If a SAR is registered, the principal investigator will notify the study coordinator within 24 hours and later the National Center for Pharmacovigilance of the Agency for Medicinal Products and Medical Devices of the Republic of Slovenia and the Ethics Committee of the Institute of Oncology Ljubljana.

### Confidentiality

On all documents collected for data analysis, patients will be identified by code only. The log of subject identification data will be maintained by the principal investigator. The name or other information that might reveal the identity of the patient will not be included in any document that leaves the research center and will not be used for clinical research data analysis. As a research participant, the patient has the right at any time to obtain information about the personal data collected and processed by the research provider, to request their correction or deletion, and to complain to the supervisory authorities, i.e. the Information Commissioner of the Republic of Slovenia and the Data Protection Commissioner of the Institute of Oncology Ljubljana.

### Data Monitoring Committee

As a body independent of studies, sponsors and competing interests, the Ethics Committee of the Institute of Oncology Ljubljana is responsible for reviewing and monitoring all ongoing studies conducted at the clinic. Each year during the study, the principal investigator prepares an annual report on the progress of the study, registered adverse effects and SAEs, which is evaluated by the aforementioned committee.

### Availability of data and materials

Electronic study records will be stored on the hospital server and will be accessible via password-protected network computers. Data will be retained for up to 10 years, after which they will be deleted.

### Statistical methods

Since this is an exploratory study, no formal sample size calculation was performed.

Data will be managed in databases with Excel spreadsheets and analyzed with statistical analysis software such as SPSS, GraphPad Prism, or similar tools. Changes in absolute values and percent deviations from baseline salivary flow rate values before and after the procedure will be analyzed and compared at different time points within the intervention group; baseline values will also be compared with those of the control group. Numeric variables will be compared with paired t tests or nonparametric tests if the assumptions for parametric tests are not met.

The scores of the individual items of the VAS questionnaire and the Xerostomia questionnaire will be summed, and the total score is linearly transformed into a scale from 0 to 100.^29,30^ Scoring and interpretation of the EORTC QLQ-H&N35 questionnaire will be performed according to EORTC guidelines.^31^

Comparisons of MRI, ultrasound, scintigraphy, and analysis of core-needle biopsies taken before and after procedure will be descriptive in nature.

## Discussion

The aim of this phase I study is to evaluate the safety and preliminary efficacy of treatment of xerostomia after irradiation of patients with oropharyngeal carcinoma with allogeneic MSCs derived from umbilical cord tissue. These cells are characterized by a high rate of stemness and marked immunomodulatory activity. Compared with autologous MSCs or allogeneic MSCs from adipose or bone marrow tissue, their collection and processing are less invasive and simpler.^32^ If our hypothesis is confirmed, our results will make an important contribution to the optimization of MSC treatment of xerostomia after radiation and will be used in the design of the next clinical trials.

Treatment of xerostomia after radiation with MSCs is one of the newest therapeutic approaches. Due to the complex pathophysiology of radiation-induced salivary gland hypofunction, which also involves immune mechanisms, MSCs represent an interesting therapeutic agent because they have both immunoregulatory and regenerative effects.^33^ The mechanism of action of MSCs is therefore multifaceted and well suited for the treatment of diseases with a complex etiologic background. Although MSCs are naturally present in affected tissues, their numbers are significantly reduced after irradiation.

The first reports on the treatment of xerostomia with MSCs in animal models appeared about ten years ago. In irradiated mouse models, systemic therapy with adipose-derived MSCs was shown to improve salivary flow.^34^ Following therapy, there was an increase in mucin secretion and amylase production.^35^ In addition, MSC therapy affected the architecture of the gland by increasing the number of functional acini and improving the microvascular structure. Compared with the untreated group, MSC-treated mice had a lower number of apoptotic cells, confirming the known anti-apoptotic and mitogenic effects of MSCs.^35,36^

There is less clinical evidence for the efficacy of xerostomia therapy with MSCs compared with some other indications, e.g., orthopedics or hematology. However, significant progress has been made in recent years. In 2018, Grønhøj *et al*. were the first to publish the results of a phase I/II clinical trial evaluating the safety and efficacy of therapy for xerostomia after RT with autologous MSCs.^37^ They performed a randomized, placebo-controlled trial in 30 patients with squamous cell carcinoma of the oropharynx who had been cured with (chemo)radiotherapy two or more years ago. MSCs were applied to both submandibular glands under ultrasound guidance. The amount of cells (drug dose) was calculated using available preclinical data, taking into account gland volume in humans. The maximum dose of MSCs was approximately 4.5×10^7^ MSCs per gland (2.86×10^5^ – 2.86×10^6^ MSCs/cm^3^ of gland). No side effects were observed during the study and up to two years after treatment. Compared to the placebo group, the treated group showed a statistically significant increase in unstimulated salivary flow both one month (33% increase, p=0.048) and four months after treatment (50% increase, p=0.003). Four months after MSC application, treated patients reported significant improvement in xerostomia symptoms (VAS questionnaire) compared to the placebo group. In a subsequent report with a median follow-up of 3.6 years after treatment, the authors reported no serious adverse events associated with the intervention, but maintained a positive clinical effect of MSC treatment.^38^

In addition, Lynggaard *et al*. published encouraging results on the treatment of radiation-induced xerostomia with allogeneic adipose-derived MSCs.^39^ The use of allogeneic MSCs offers numerous logistical advantages: surgical intervention is no longer required to obtain the starting material, and the quality of the final preparations can be better controlled.^40^ In this study, 25×10^6^ MSCs were inserted into each submandibular gland and 50×10^6^ MSCs were inserted into each parotid gland in ten patients; the follow-up period was four months. During this period, an increase in mean unstimulated salivary flow was measured from an initial 0.13 ml/min to 0.18 ml/min. Stimulated salivary flow also increased after therapy from 0.66 ml/min to 0.75 ml/min. Patients’ subjective perception of an improvement in xerostomia was confirmed by questionnaire results, and no serious adverse events were reported during the study period.^39^

Recently, a dose-escalating phase I study protocol (3 + 3 design) was published for the treatment of radiation-induced xerostomia in patients with head and neck cancer with interferon-gamma-stimulated autologous bone marrow stromal cells.^41^ The results of this study are not yet available. However, in a pilot study, the same group has already used MSC prepared in this manner in six patients who received a single injection of 10×10^6^ MSC into a single mandibular salivary gland and found that the therapy was well tolerated and showed a trend toward improvement in salivary volume and quality of life.^42^

To our knowledge, there has been no research using allogeneic MSCs from umbilical cord tissue. We believe that this may have several advantages. First, the collection of umbilical cord tissue is noninvasive for both the patient and the donor. The relative ease of obtaining umbilical cord tissue allows for greater flexibility in cryobanking “off the shelf” MSC products. This is also accelerated by the biological advantages of MSCs from umbilical cord tissue compared with, for example, bone marrow or adipose tissue, namely their high proliferation rate, smaller cell size, and lower rate of senescence during cell culture, which allows for higher cell yield during manufacturing.^32,43^ Last but not least, umbilical cord tissue-derived MSCs have been reported to have excellent immunomodulatory potential, very low immunogenicity, and documented regenerative properties that may outperform tissue-derived MSCs in certain aspects.^44,45^ Because this is an academic clinical trial focusing on a very limited number of patients, we chose a single dose consistent with previous report of safe use of allogeneic adipose-derived MSCs for the treatment of xerostomia.^39^ We believe that this is also a safe dose, both because of the small cell size of MSCs from umbilical cord tissue and because of their low immunogenicity profile.^46^

Because this is the first attempt to evaluate the use of MSCs from umbilical cord tissue for the treatment of xerostomia, we made every effort to design our study to be as similar as possible to the designs of previously published studies. For example, we used comparable study inclusion criteria (oropharyngeal cancer, at least 2 years after RT, and no evidence of recurrence) and exclusion criteria, examinations (unstimulated and stimulated salivary flow measurements with salivary composition analysis; biopsy, MRI, and US of the salivary glands; questionnaires on xerostomia and quality of life), and time points for assessing treatment effect (4 weeks and 4 months after procedure).^39,41,47^ The nuclear medicine studies in our protocol aim to provide additional definition of the effect of the administered therapy. This should enable us to make a more credible comparison of our results with those of other authors, which will undoubtedly help to evaluate the potential of our approach compared with previously studied methods for the treatment of xerostomia after irradiation with MSCs.

## Conclusions

The treatment of xerostomia after irradiation with MSCs represents a promising new therapeutic method, which is expected to trigger the regeneration of the glandular tissue and improve its function, with a positive impact on patients’ quality of life. Moreover, a crucial aspect is the fact that no serious adverse effects related to MSC therapy have been observed up to 3.6 years (median) after this type of treatment in the clinical trials performed so far.^32^ We expect that the results of our study will contribute significantly to a deeper understanding of the effects of treatment with allogeneic MSC from umbilical cord tissue and optimize this therapy. The research results will not only be valuable from a scientific or academic point of view but will also have practical and clinical significance.
